# Genome Editing: A Promising Approach for Achieving Abiotic Stress Tolerance in Plants

**DOI:** 10.1155/2022/5547231

**Published:** 2022-04-15

**Authors:** Navdeep Kaur, Shubham Sharma, Mirza Hasanuzzaman, Pratap Kumar Pati

**Affiliations:** ^1^Centre for Agricultural Research and Innovation, Guru Nanak Dev University, Amritsar, 143005 Punjab, India; ^2^Department of Biotechnology, Guru Nanak Dev University, Amritsar, 143005 Punjab, India; ^3^Department of Agronomy, Faculty of Agriculture, Sher-e-Bangla Agricultural University, Dhaka 1207, Bangladesh

## Abstract

The susceptibility of crop plants towards abiotic stresses is highly threatening to assure global food security as it results in almost 50% annual yield loss. To address this issue, several strategies like plant breeding and genetic engineering have been used by researchers from time to time. However, these approaches are not sufficient to ensure stress resilience due to the complexity associated with the inheritance of abiotic stress adaptive traits. Thus, researchers were prompted to develop novel techniques with high precision that can address the challenges connected to the previous strategies. Genome editing is the latest approach that is in the limelight for improving the stress tolerance of plants. It has revolutionized crop research due to its versatility and precision. The present review is an update on the different genome editing tools used for crop improvement so far and the various challenges associated with them. It also highlights the emerging potential of genome editing for developing abiotic stress-resilient crops.

## 1. Introduction

The world population is anticipated to reach up to 10 billion by the end of the year 2050. In this scenario, the production of food crops is required to be increased by 60% in the coming years for ensuring global food security [1, 2]. However, abiotic stresses including salinity, drought, and extreme temperatures are some of the primary constraints that significantly hamper the production and yield of crop plants [3, 4]. They are responsible for almost 50% yield loss of crop plants resulting in $14–19 million annual economic loss worldwide [5, 6]. The effect of these stresses is further expected to aggravate due to the climatic uncertainties in near future [3, 7]. Thus, management of abiotic stresses in different crops is one of the prime aims of researchers for ensuring sustainable agriculture.

Researchers have endeavored to develop various approaches from time to time for achieving abiotic stress tolerance in plants [8–11]. The most conventional means involves crossing of two parental plants for producing a progeny plant having desired characteristics. However, this approach has met only limited success due to the complexity of traits associated with abiotic stress tolerance [12–14]. In the past decade, with the advancement in the field of genomics and molecular biology, the emergence of the genetic engineering approach has provided great opportunities for increasing the stress tolerance of plants [15, 16]. In this strategy, abiotic stress-tolerant crops are generated by mobilizing genes or their regulating elements into the genome of interest [17]. However, the worldwide use of genetically modified crops is restricted due to the various health and environmental concerns associated with their use [18, 19]. Thus, to fulfill the food demand of the growing world population, it is imperative to devise novel and potent strategies that can help in the production of abiotic stress-resilient crops with better growth and yield. In this scenario, the emergence of genome editing has revolutionized the field of plant science and agriculture [1, 20].

Genome alteration is a natural phenomenon that exists on earth for thousands of years. Plants with specific genetic variations have been selected over others through evolution since long. Almost all the crops grown nowadays have sustained considerable genetic variations in the past [21, 22]. Modern corn grown worldwide that is substantially different from its wild ancestor teosinte is such an example. However, our ancestors relied upon the naturally occurring genome variations/mutations to improve the various crop traits [22]. Later in the 20^th^ century, when it was established that DNA forms the genetic basis of life and controls the phenotype of any organism, researchers introduced changes in the genome using chemical mutagens and radiations. This type of mutagenesis was very resourceful for the mutational breeding that has shown significant success in the Green Revolution of the 1970s [22, 23]. However, the drawbacks of random mutagenesis and stringent selection procedures for mutants have generated a pressing need to develop new approaches that can alter the DNA sequence in a specific manner [24]. In this context, genome editing offers a precise modification of the target genome in an integration-free mode [9]. The concept of genome editing was conceived by Capecchi in the 1980s [22, 25]. In this approach, the genetic material can be removed, altered, or added at specific loci within the genome [26]. During this process, double-stranded (ds) DNA breaks (DSB) are created in a sequence-specific manner using sequence-specific endonucleases (SSEs). SSEs are expressed within the cell transiently either using mRNA that quickly degrades once its job is done or in the form of a protein that is not transferred to future generations [27–31]. The gaps generated during this process are then repaired by host DNA repair machinery via nonhomologous end-joining (NHEJ) and homologous recombination (HR). NHEJ system operates through two pathways including Ku-dependent and Ku-independent [32, 33]. During the Ku-dependent pathway, Ku proteins KU70/80 bind to the ends of ds breaks at the target site. This binding leads to the recruitment of repair machinery comprising of ligase IV enzyme along with its cofactors resulting in sealing the gaps [32]. However, in the Ku-independent pathway, the broken ends of the DNA sequence are resected to generate a single-stranded overhang. These overhangs are then aligned with the help of 5-25 bp long microhomologous sequences. The resulted gaps are later filled with the DNA polymerase and nicks are ligated using DNA ligase [34]. NHEJ repair system is error-prone and may result in deletions or insertions in the target genome. On the contrary to this, the HR repair system is error-free as in this pathway, a DNA template is used to accurately replace the nucleotides and thus HR results in insertions or replacements in the target genome [33]. Genome editing has been used for improving the various traits in plants for almost a decade; however, in the past few years, it has progressed to a phenomenal degree of success because of the development of simpler genome editing tools. Although a few earlier research papers have dealt with the CRISPR/Cas-mediated abiotic stress amelioration in plants, yet there is scarcity of review articles that holistically discuss the potential of different available genome editing tools to overcome the problem of abiotic stress in plants. The present review is an update on the various types of genome editing tools used for crop improvement and the advancements on their potential to impart abiotic stress tolerance. In addition, it also highlights the different challenges that hurdles the harnessing of available genome editing tools to their full potential for improving the abiotic stress resilience of plants.

## 2. Different Plant Genome Editing Techniques

Plant genome editing techniques have been classified into four major types to date based on onsite-specific endonucleases. These consist of meganucleases, zinc-finger nucleases (ZFNs), transcription activator-like effector nucleases (TALENs), and clustered regularly interspaced short palindromic repeats (CRISPR)/CRISPR-associated protein 9 (Cas9) (Table 1) [20, 35].

### 2.1. Meganucleases

Meganucleases are the endonucleases that can recognize and cut larger DNA sequences (>12 bp) in a sequence-specific manner [36, 37]. They are reported to occur in a range of organisms including archaebacteria, bacteria, algae, fungi, yeast, and some of plant species. They were initially discovered in the 1980s and were later extensively characterized in the early 1990s in mammalian cells [38]. Due to the presence of long recognition sites, their target site is expected to occur only once in a large genome. Moreover, meganucleases can endure mild polymorphisms at the target site [38]. They are also known as homing endonucleases and are assumed to act as parasitic elements of the host genome. Meganucleases create ds DNA breaks in the host genome at a specific position and then propagate themselves in the given genome using the homologous repair system of the host; however, their exact role is still not clear [39]. Meganucleases have been classified into five families on the basis of their sequence and motifs present in their structure. These include LAGLIDADG, PD-(D/E) XK, His-Cys box, GIY-YIG, and HNH [39, 40]. Among these, the LAGLIDADG meganuclease (LMN) family has been extensively used for genome editing. Its name is derived from the sequence of the major motif present in the structure of this family of proteins [37, 39]. LMNs are usually expressed in the chloroplast and mitochondria of unicellular eukaryotes. Most of these endonucleases exist as dimeric proteins and exhibit two roles including RNA maturase activity responsible for their own intron splicing and a specific endonuclease activity that result in the cleavage of the exon sequence [38]. Meganucleases generate ds breaks which are repaired using the NHEJ system resulting in error-prone deletions or micro insertions at the target site [38, 41]. Among various members of this family, the most widely used for genome editing are I-SceI and I-CreI. *I-SceI* gene is localized in the 21S rRNA encoding gene of mitochondrial DNA of *Saccharomyces cerevisiae*. It identifies 18 bp long sequence 5′-TAGGGATAACAGGGTAAT-3′ and is known as the “gold standard” in the field of genome editing due to its specificity and activity. *I-CreI* was identified in the chloroplast of unicellular algae *Chlamydomonas reinhardtii* and is localized in the 23S rRNA gene. It exists as a homodimer and recognizes 22 bp long target sequence 5′-CAAAACGTCGTGAGACAGTTTG-3′. The catalytic region of I-CreI consists of dual aspartic acid residues which participate in cleaving the DNA [38, 42]. Meganucleases have been used as scaffolds for engineering endonucleases in customized gene therapy [38]. However, due to the difficulties involved in the process of reengineering meganucleases to target novel DNA sequences, its use in genome editing has been limited [43, 44].

### 2.2. Zinc-Finger Nucleases

Zinc-finger nucleases (ZFNs) are the artificially synthesized endonucleases that can cleave ds DNA in a sequence-specific manner [45, 46]. They can be used to introduce a wide range of genetic modifications including insertions, deletions, translocations, inversions, and point mutations in the target genome [47]. Thus, ZFNs have been used in the majority of genome editing experiments in various organisms to date [47, 48]. They consist of two domains, viz., the first DNA binding domain and another nuclease domain. DNA binding domain is composed of 3- to 6-zinc finger repeats, and each of these repeats can recognize a 9-18 bp long nucleotide sequence. The other domain comprises of a restriction enzyme *Flavobacterium okeanokoites*I (*Fok*I) and is involved in the cleavage of DNA [48]. *Fok*I enzyme needs to dimerize to function accurately, and thus for cleaving each target sequence, a pair of ZFNs is required [46, 49]. To cleave the target DNA sequence, each of the two ZFN molecules binds to the opposite DNA strands for proper dimerization. Earlier, homodimeric *Fok*I domains were used in the ZFNs that sometimes resulted in the formation of unwanted dimeric species with similar monomeric ZFNs. However, in the past few reports, heterodimeric *Fok*I domains have been designed for ZFNs, which substantially reduces the generation of unnecessary homodimers of the *Fok*I domain, thus improving their specificity [47]. Once dimerized, *Fok*I cleaves the target DNA sequence introducing DSB which is repaired either by NHEJ or HR. In the presence of a homologous template sequence, HR machinery is used; however, in its absence, the NHEJ mechanism is used resulting in frameshift mutations due to the insertion or deletion of nucleotides. ZFNs can be designed using different protein engineering methods to virtually target any novel stretch of DNA [50]. ZFNs with improved specificity and activity have been engineered to successfully generate knockouts to remove the function of the gene and also to incorporate gain of function mutations [51].

### 2.3. Transcription Activator-Like Effector Nucleases

Transcription activator-like effector nucleases (TALENs) are the engineered restriction enzymes that are designed to cut specific DNA sequences [52]. They also consist of two domains like ZFNs, the TALE DNA binding domain and the DNA cleaving domain [46]. TALE DNA binding domain is made up of TAL effector (TALE) proteins. TALEs are the naturally occurring transcription factors secreted by the Xanthomonas genus of plant pathogens upon infection [53]. They are made up of 33-34 amino acids conserved repetitive sequences with variation at 12^th^ and 13^th^ position referred to as repeat variable residues (RVDs). These RVDs are solely responsible for the recognition of specific DNA sequences that act as a substrate for TALEN molecules. In most of the customized TALENs, RVDs consist of histidine-aspartic acid (HD) for cytosine, asparagine-asparagine (NN), asparagine-isoleucine (NI) for adenine, asparagine-histidine (NH), asparagine-asparagine (NN), asparagine-lysine (NK) for guanine, and asparagine-glycine (NG) for thymine [54, 55]. It was revealed from the crystal structure of TALE that the RVD residue at the 12^th^ position is involved in making a stabilized contact with the backbone loop and the 13^th^ position RVD is involved in forming a base-specific contact with the target DNA [56, 57]. Researchers have also examined the genomes of other organisms for discovering TALE-like proteins. In one such attempt, Ralstonia TALE-like proteins (RTLs) have been isolated from the bacteria *Ralstonia solanacearum*. RTLs are structurally similar to TALEs; however, they possess different RVD residues [54]. DNA cleavage domain of TALENs is composed of *Fok*I enzyme-like ZFNs. Thus, TALEN also needs to dimerize to act on their target sites [47].

Due to specificity in DNA binding, TALEs can be used for synthesizing epigenetic and transcriptional regulators [22]. It has been proposed that the activity of the TALEN molecule is regulated by the flanking amino acids present on the N and C terminal of the DNA recognition domain [58]. The removal of a few amino acids from both of these ends provides stability to the proteins by facilitating proper folding. The resulting truncated versions of TALEN molecules have also been found to show better activity [59–61]. For creating such customized TALEN molecules, a ligation-based “Golden Gate System” has been the method of choice of the researchers [62–64]. However, the more advanced “Platinum TALEN and Platinum Gate System” has also been developed that is used to incorporate mutations in eukaryotes [33].

### 2.4. Clustered Regularly Interspaced Short Palindromic Repeats/CRISPR-Associated Protein

The Clustered Regularly Interspaced Short Palindromic Repeat/CRISPR-Associated System (CRISPR/Cas) is the recent breakthrough approach in the field of genome editing that has become a leading research tool for targeted mutagenesis in eukaryotes [65, 66]. This tool has been espoused from the defense machinery of bacteria. Different archaeal and bacterial species use CRISPR/Cas system to protect themselves from the invading viruses [67]. Most of the archaea and almost 40% of the different bacterial species sequenced genomes possess CRISPR/Cas system that can degrade RNA, DNA, or both [68, 69]. Whenever a phage infects a bacteria having CRISPR/Cas defense mechanism, the host bacterium degrades the invading DNA/RNA in three phases, viz., adaptation, biogenesis, and immunity/interference phase. In the adaptation phase, a fragment of pathogenic DNA is acquired by the host CRISPR array which is then transcribed into mature RNA also called as cr RNA in the biogenesis phase. These cr RNAs are later used by the nuclease enzyme Cas as a guide RNA to degrade the target sequence in the immunity phase [70, 71]. However, for the action of Cas endonuclease, the target site must contain a short DNA sequence known as “protospacer-adjacent motif” (PAM) downstream to the given target site, and it should be compatible with the given Cas protein [72–76].

The CRISPR system is adopted from the bacteria *Streptococcus pyogenes* that use Cas9 nuclease, and a single-guide RNA molecule has been most widely exploited for genome editing [67, 75]. For this purpose, 20-22 nucleotides long target-specific small guide RNAs (sg RNA) are synthesized which recognize their target and base pairs with them using Watson and Crick base pairing [77].Cas9 enzyme later cuts the recognized target site at specific positions, and the resulting gaps are filled using an HR repair system [77]. Initially, Cas9 isolated from *Streptococcus pyogenes* was used for most of the genome editing experiments; however, in the past few years, several variants of Cas9 have been generated through improvements in the wild-type Cas9 [78]. Apart from Cas9, various other types of Cas including Cas3, Cas12, and Cas13 have been recently explored for their potential application in genome editing [79]. Among these, Cas12 is the most preferred choice for genome editing of plants after Cas9 [80]. In contrast to other genome editing tools, this system is simpler, versatile, and inexpensive and thus has been widely used for improving the different traits in almost all the kingdoms of life [67]. Moreover, the past few years are also marked as “CRISPR craze” years by the researchers due to the immense use of this approach. Further, the ability of CRISPR/Cas to offer multiplex genome editing makes it a method of choice by researchers [81, 82].

## 3. Genome Editing: A Boon for Generating Abiotic Stress-Resilient Plants

**Figure 1:**
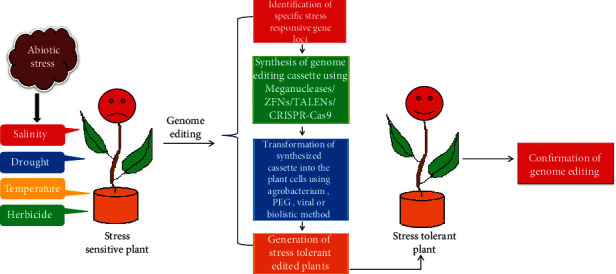
Schematic representation of the process of genome editing for generating abiotic stress-tolerant plants.

To sustain the increasing global population, it is imperative to escalate the production of food crops exponentially. However, the susceptibility of plants to an array of abiotic stresses is a major challenge to achieving this goal [83]. Earlier, many abiotic stress-tolerant crop plants have been developed using conventional marker-assisted breeding. However, this approach takes almost a decade to successfully develop abiotic stress-resilient crops due to intensive screening and backcrossing procedures [84]. The stress-tolerant plants developed through genetic engineering have shown promising results, but there are number of hurdles in their commercialization. Hence, in this scenario, genome editing appears to be a sophisticated approach to develop abiotic stress-resilient crops in the future as it offers precise manipulation of various gene loci in comparatively lesser time, thus reducing the cost of crop improvement programs (Figure 1) [85]. Genome editing has already been successfully used to improve the various traits of plants including their nutritional value and yield [22]. On the contrary, there are only a few reports on its use for generating abiotic stress-tolerant plants. However, due to the development of highly efficient genome editing tools like CRISPR/Cas, it can provide ample opportunities to improve the abiotic stress resilience of plants. Moreover, it can be used to understand the role of various stress signaling cascades in abiotic stress adaptation [77]. Here, we discuss the key advances in the use of genome editing for improving the abiotic stress resilience of plants (Table 2).

### 3.1. Salinity Stress

Salt stress is a major abiotic stress that is responsible for a significant reduction in the global productivity of different crops [86, 87]. It affects almost 80 million hectares of global irrigated land, and this area is further expected to increase due to changing global climate [88]. Although the potential of genome editing in crop improvement is well-realized, still there are only a few reports on the successful use of this approach for achieving salinity stress tolerance in plants. In one such report, the *OsRR22* gene that encodes for a transcription factor (TF) involved in the regulation of signaling as well as the metabolism of cytokinins in plants has been edited in rice to confer salt stress tolerance [89, 90]. Earlier, it was reported that loss of function of this gene can significantly hamper the cytokinin pathway, thus conferring salt stress resistance in plants [89]. The rice knockouts of *OsRR22* produced using CRISPR/Cas9 performed better under salt stress [90]. Further, genome editing has been used to understand the molecular regulation of various genes involved in abiotic stress adaptation. RAV2 is a TF that belongs to AP2/ERF family and plays a critical role in abiotic stress tolerance [91]. However, its regulation in response to salt stress was not elucidated. Genome editing of the promoter sequence of the *RAV2* gene using CRISPR/Cas9 revealed that the GT-1 element that is localized at the −664 position relative to the putative start site of translation of *RAV2* is critical for its induction in response to salt stress [91]. In another report, the loss of function mutants of the *SAPK2* gene that plays a key role in ABA signaling during osmotic stress showed increased sensitivity towards salt stress stimuli emphasizing the involvement of *SAPK2* in salt stress adaptation [92]. Recently, the *drought and salt tolerance* (*DST*) gene of rice has been mutated using CRISPR/Cas for improving salt/drought stress tolerance in plants. The resultant *dst*^*∆184*–*305*^ mutant plants exhibited a reduction in stomatal density and showed enhanced levels of water retention in leaves. The decrease in stomatal density was linked to the downregulated expression of different genes involved in the development of stomata including *MUTE*, *ICE1*, and *SPCH1* in mutant plants [93]. Moreover, in tomatoes, alteration of 8CM and PRD domains of hybrid proline-rich protein 1 (HyPRP1) with the advent of CRISPR/Cas resulted in improved salinity stress tolerance [94]. Further, a micro-RNA encoding gene *OsmiR535* that regulates the expression of abiotic stress-responsive genes at the posttranscriptional level was mutated using CRISPR/Cas. The resulting knockout *osmir535* rice mutant plants showed better performance upon exposure to salt stress as compared to the control plants [95]. From these reports, it is clearly evident that genome editing can impart salinity stress resistance in plants. Thus, in future, research efforts should be focused on targeting the key salinity stress response genes (especially those that can regulate multiple stress responses such as transcription factors) with the advent of genome editing.

### 3.2. Drought Stress

Drought is another multidisciplinary stress that affects the growth of plants at various morphological, physiological, and biochemical levels [96]. The effect of this stress is further expected to increase in the coming years due to climate change [97]. Genome editing has been successfully employed for improving the drought tolerance of different crops. *OPEN STOMATA 2* (*OST2*) gene encodes for an H^+^-ATPase and is involved in creating proton gradients in plant cells. Precise modification of this gene via CRISPR/Cas9 has been reported to modulate stomatal closing in response to water-deficient conditions, thus conferring drought stress tolerance [98]. The loss of function *sapk2* mutant rice plants produced using CRISPR/Cas exhibited more sensitivity against drought stress that was linked to the modulation of the expression of several genes that acts downstream of the SAPK2 including *OsOREB1*, *OsRab21*, *OsRab16b*, *OsLEA3*, *OsbZIP23*, *OsSLAC1*, and *OsSLAC7* genes [92]. *ARGOS8* is yet another drought stress-responsive gene modulated using genome editing. It is a negative regulator of the ethylene signaling pathway, and its increased expression is known to confer drought stress resistance in plants [99, 100]. The replacement of the promoter sequence of *ARGOS8* using CRISPR/Cas9 with GOS2 promoter resulted in the increased ubiquitous expression of this gene in maize, thus enhancing their resistance towards drought stress [101]. Further, it has been realized that genome editing can be successfully used for improving the different traits of diploid plants; however, its implementation on the polyploid and complex genomes is a major challenge. Recently, CRISPR/Cas9 has been used to edit the *TaDREB2* gene that encodes for a dehydration responsive element binding protein in the protoplast of wheat to generate drought stress-resistant plants that open new opportunities to improve the stress resistance of polyploid plants using genome editing [102]. Moreover, editing of the trehalase gene that plays a key role in trehalose catabolism using CRISPR/Cas improved the drought tolerance in *Arabidopsis thaliana* [103]. Trehalase enzyme catalyzes the only reaction of trehalose catabolism in plants and results in the hydrolysis of trehalose into D-glucose molecules. The plants with edited substrate-binding domain of trehalase enzyme showed drought-tolerant phenotypic traits [103]. Besides, the alteration of *DST* and *miR535* genes using CRISPR/Cas leads to improved drought tolerance in addition to salinity stress acclimatization in rice [93, 95]. Overall, the genome editing has played a pivotal role in the identification and characterization of critical drought stress-responsive genes that can be targeted in future crop improvement programs using various available genomics approaches.

### 3.3. Temperature Stress

Plants grow at an optimum temperature and thus any increase or decrease in this temperature can seriously hamper their growth and yield [104, 105]. As the temperature of the earth is continuously increasing due to global climate change, the development of novel strategies to combat the effects of temperature extremities in plants is a major challenge [106]. Researchers have attempted to improve the survival of different plants in response to temperature stress using genome editing. The *9-cis-EPOXYCAROTENOID DIOXYGENASE4* (*NCED4*) gene knockout mutants of lettuce plants produced using CRISPR/Cas9 has been reported to show better thermotolerance at the germination stage [107]. CRISPR/Cas9 technology has also been used to edit two genes *TIFY1a* and *TIFY1b* in rice to decipher their precise role in cold stress tolerance [108]. Moreover, the CRISPR/Cas-mediated editing of the *Ann3* gene of rice revealed its key role in cold stress adaptation [109]. Similarly, mutagenesis of tomato *C-repeat binding factor 1* (*CBF1*) gene unraveled the importance of this gene in imparting cold stress tolerance in tomatoes. The *cbf1* mutant tomato plants were observed to be more sensitive towards the stress conditions and accumulated more indole acetic acid along with enhanced electrolyte leakage [110]. Further, in a recent study, three rice genes, viz., *OsPIN5b*, *GS3*, and *OsMYB30*, were altered using CRISPR/Cas9 resulting in improved cold stress tolerance along with better yield [111]. Despite these few reports, the potential of genome editing has not been fully explored for conferring temperature stress resistance in plants. Thus, conscious efforts are much required for the identification of key genes that regulate temperature stress responses and their modulation using genome editing for protecting the plants against heat/cold injury in the era of global climate change.

### 3.4. Herbicide Stress

The uncontrolled growth of herbs imposes a major threat to the annual yield of many crops, and thus, herbicides are routinely used in agriculture to control the growth of weed plants. However, during this process, the use of herbicides also imposes a considerable menace on the growth of nontarget plants [112]. Thus, to mitigate the effects of herbicides, it is imperative to develop herbicide-resistant plants. Genome editing has been successfully used for imparting herbicide resistance in plants. Different genes have been altered using ZFNs, TALENs, and CRISPR/Cas for imparting herbicide resistance to plants, and among these, *ALS* and *EPSPS* are the major ones. *ALS* gene encodes for an enzyme acetolactate synthase that catalyzes the key step in the biosynthetic pathway of branched amino acids including leucine, isoleucine, and valine [113, 114]. A range of herbicides, such as the imidazolinones, sulfonylureas, triazolopyrimidines, sulfonylamino-carbonyl-triazolinones, and pyrimidinylthio (or oxy)-benzoates, have been reported to inhibit the activity of ALS in plants [115, 116]. In 2009, this gene was precisely modified using the ZFN genome editing tool. The resulting alteration made the tobacco plants resistant to sulfonyl urea herbicides [117]. Later, the *ALS* gene was altered in a range of crop plants such as soybean, maize, potato, and watermelon using TALENs and CRISPR/Cas9 tools [118–121]. *EPSPS* is another gene that encodes for an enzyme 5-enolpyruvylshikimate-3-phosphate that plays a critical role in the biosynthetic pathway of essential aromatic amino acids via the shikimate pathway [122]. This enzyme is highly sensitive to a routinely used herbicide glyphosate [123]. The alteration of two nucleic acid residues in the binding site of glyphosate-EPSPS using CRISPR/Cas9 has resulted in the modified genotypes of flax and rice with improved tolerance towards glyphosate [124, 125]. In addition to *ALS* and *EPSPS*, there are few reports on the alteration of *Acetyl-CoA carboxylase* (*ACCase*) gene for achieving herbicide resistance in the target plant using genome editing [126]. ACCase plays a pivotal role in fatty acid biosynthesis and herbicides including cyclohexanedione (CHD), aryloxyphenoxypropionate (APP), and phenylpyrazoline (PPZ) act on this enzyme. This gene has been altered using CRISPR/Cas in rice and wheat for achieving herbicide resistance [127, 128]. Although there are many success stories where genome editing has successfully addressed the menace of herbicide stress, but in these reports, resistance has been primarily achieved only for ACCase-inhibiting, ALS-inhibiting, and glyphosate herbicides. Thus, future research progress on the development of herbicide-resistant plant varieties against other classes of herbicides including protoporhyrinogen oxidase inhibiting and 4-hydroxyphenyl pyruvate dioxygenase herbicide is much warranted [126].

## 4. Challenges Associated with Genome-Edited Crops

Genome editing offers a robust method to accomplish genetic modifications in an integration-free manner using SSEs [129]. However, there are some challenges associated with this approach that needs to be addressed for its successful implementation in crop improvement programs. The major concern in this regard is the occurrence of off-target mutations [130]. It has been reported that genome editing tools especially CRISPR/Cas9 often recognize an imperfect match thus cutting the nucleotide sequences nonspecifically. Although, these off-target mutations are expected to be segregated during stringent breeding/selection process in different crop improvement programs, still they impose a major concern on the efficient use of genome editing in plants [130]. To solve this issue, researchers have designed various softwares including CRISPR-GE and CRISPR-P that can aid up in accurately designing the guide RNA [131, 132].

The commercialization and regulation of genome-edited crops are also highly debated by different regulatory authorities and scientific communities [133–136]. An internationally accepted set of regulations for genome-edited crops is still lacking [130]. The major concern in their regulation is the dilemma that whether such crops should be considered under the genetically modified organism (GMO) regulations or not. In this context, USDA has exempted the genome-edited crops from GMO regulation in 2012 and has approved the cultivation and commercialization of waxy corn and mushrooms edited using the CRISPR/Cas system [137–139]. However, the European Court of Justice (ECJ) has included the genome-edited crops under the GMO regulations that can hinder their commercialization in Europe and may also affect agricultural trade with European countries [140].

## 5. Conclusion

Genome editing is a valuable tool for crop improvement programs due to its efficiency, simplicity, and specificity [141]. It has opened up new opportunities in the field of functional genomics and plant breeding. Preliminary studies have established that genome editing is a suitable approach for producing abiotic stress-resilient crops in the future to solve the problem of hunger. However, the various technological and regulatory hurdles are still required to be solved for reshaping global agriculture using genome editing.

## Figures and Tables

**Table 1:** Comparison of different types of genome editing tools [142–145].

Feature	Meganucleases	ZFNs	TALENs	CRISPR/Cas
Target site length	12-40 bp	18-36 bp	28-40 bp	20-22 bp
Recognition	Protein recognize DNA	Protein recognize DNA	Protein recognize DNA	RNA protein complex recognize DNA
Nuclease protein	I-SceI	FokI	FokI	Cas
Dimerization	Not required	Required	Required	Not required
Repair events	HDR	NHEJ	HDR	NHEJ
Efficiency	Moderate	Low	Moderate	High
Specificity	High	Moderate	High	Low
Multiplexing	Challenging	Challenging	Challenging	Feasible
Cost	High	High	Moderate	Low
Ease of engineering	Low	Low	Moderate	High

**Table 2:** Summary of the different genes used for achieving abiotic stress tolerance in plants using genome editing approach.

Plant	Targeted Gene	Role of the Gene	Method Used	Stress	Reference
Rice, Wheat	*ACCase*	Fatty acid biosynthesis	CRISPR/Cas	Herbicide	[131, 132]
Tobacco, Maize, Rice, Soyabean, Potato,	*ALS*	Biosynthesis of branched amino acids	ZFN, TALENS, CRISPR/Cas	Herbicide	[115–118]
Rice, Flax	*EPSPS*	Biosynthesis of essential aromatic amino acids	CRISPR/Cas	Herbicide	[122, 123]
Arabidopsis	*OST2*	a H^+^-ATPase	CRISPR/Cas	Drought	[96]
Rice	*RAV2*	Abiotic stress responsive transcription factor	CRISPR/Cas	Salinity	[89]
Maize	*ARG0S8*	Negative regulator of ethylene response	CRISPR/Cas	Drought	[99]
Rice	*TIFY1a*, *TIFY1b*	Cold stress responsive transcription factor	CRISPR/Cas	Cold	[106]
Rice	*SAPK2*	ABA signaling	CRISPR/Cas	Salinity/Drought	[90]
Rice	*Ann3*	Ca^2+^-dependent phospholipid-binding proteins involved in plant development stress responses	CRISPR/Cas	Cold	[107]
Wheat	*TaDREB2*	Dehydration responsive gene	CRISPR/Cas	Drought	[100]
Lettuce	*9-cis-EPOXYCAROTENOID DIOXYGENASE4* (*NCED4*)	ABA biosynthesis	CRISPR/Cas	Temperature	[105]
Tomato	*CBF1*	Cold stress responsive gene	CRISPR/Cas	Cold	[108]
Rice	*OsRR22*	Transcription factor involved in cytokinin signaling and metabolism	CRISPR/Cas9	Salinity	[88]
Rice	*DST*	Zinc finger transcription factor	CRISPR/Cas9	Salinity/Drought	[91]
Tomato	*HyPRP1*	Key role in plant developmental process and stress amelioration	CRISPR/Cas	Salinity	[92]
Rice	*miR535*	microRNA that regulates the expression of the abiotic stress-responsive gene at the post-transcriptional levels	CRISPR/Cas	Salinity/Drought	[93]
Rice	*OsPIN5b*, *GS3*, and *OsMYB30*	Panicle length regulating gene, grain size regulating gene, and transcription factor that regulates cold tolerance, respectively	CRISPR/Cas	Cold	[109]
*Arabidopsis*	*Trehalase*	Trehalose catabolism	CRISPR/Cas	Drought	[101]

## Data Availability

All data are included in the manuscript.

## Abbreviations

SSE: Sequence-specific endonucleases
ds: Double stranded
NHEJ: Nonhomologous end-joining
HR: Homologous recombination
ZFN: Zinc-finger nucleases
TALENS: Transcription activator-like effector nucleases
TALE: TAL effector
CRISPR: Clustered regularly interspaced short palindromic repeats
Cas9: CRISPR-associated protein 9
PAM: Protospacer-adjacent motif
RVDs: Repeat variable residues (RVDs)
GMO: Genetically modified organisms
USDA: United States Department of Agriculture
EU: European Union
